# Ultrathin flexible graphene films with high thermal conductivity and excellent EMI shielding performance using large-sized graphene oxide flakes†

**DOI:** 10.1039/c8ra09376h

**Published:** 2019-01-11

**Authors:** Shaofeng Lin, Su Ju, Jianwei Zhang, Gang Shi, Yonglyu He, Dazhi Jiang

**Affiliations:** Department of Materials Science and Engineering, National University of Defense Technology Changsha Hunan 410073 People Republic of China jwzhang.nudt@gmail.com jiangdz@nudt.edu.cn

## Abstract

As the demand for wearable and foldable electronic devices increases rapidly, ultrathin and flexible thermal conducting films with exceptional electromagnetic interference (EMI) shielding effectiveness (SE) are greatly needed. Large-sized graphene oxide flakes and thermal treatment were employed to fabricate lightweight, flexible and highly conductive graphene films. Compared to graphene films made of smaller-sized flakes, the graphene film made of large-sized flakes possesses less defects and more conjugated domains, leading to higher electrical and higher thermal conductivities, as well as higher EMI SE. By compressing four-layer porous graphene films together, a 14 μm-thick graphene film (LG-4) was obtained, possessing EMI SE of 73.7 dB and the specific SE divided by thickness (SSE/*t*) of 25 680 dB cm^2^ g^−1^. The ultrahigh EMI shielding property of the LG-4 film originates from the excellent electrical conductivity (6740 S cm^−1^), as well as multi-layer structure composed of graphene laminates and insulated air pores. Moreover, the LG-4 film shows excellent flexibility and high thermal conductivity (803.1 W m^−1^ K^−1^), indicating that the film is a promising candidate for lightweight, flexible thermal conducting film with exceptional EMI shielding performance.

## Abstract 2

As the demand for wearable and foldable electronic devices increases rapidly, ultrathin and flexible thermal conducting films with exceptional electromagnetic interference (EMI) shielding effectiveness (SE) are greatly needed.

## Graphical abstract

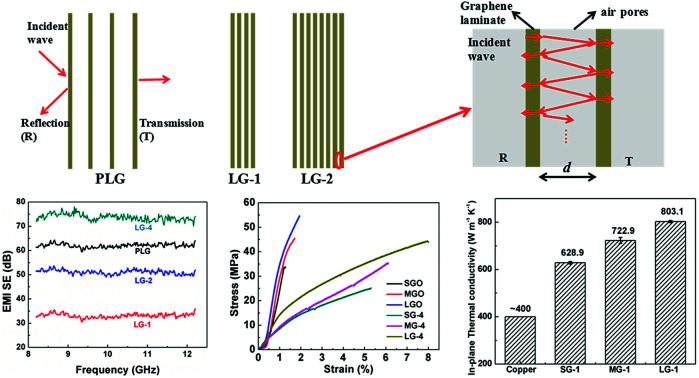

## Introduction

With the rapid development of information technology in recent years, electromagnetic (EM) pollution caused by electronic components of wearable and foldable equipments, such as flexible electrodes, storage devices, and smart sensors,^1–3^ has became a serious environmental issue. Electromagnetic interference (EMI) shielding protection is the most effective way to prevent EM pollution.^4–7^ According to electromagnetic theory, the main mechanisms to reduce undesirable EM emissions include reflection of EM radiation by impedance mismatching and absorption of the EM wave energies by dielectric or magnetic loss.^8^ Electrically conductive materials are reflective shielding materials, which could reflect most of the incident waves, due to their high electrical conductivity. For the portion of waves able to enter shielding materials, absorption loss is the dominant consumption of this EM energy, which depends on the interaction between conducting parts of shielding materials and EM waves.^7,9,10^

Hence, materials with high electrical conductivity, such as metals, carbon materials and conductive polymers, have been applied as effective EMI shielding materials. However, metal shielding materials have the disadvantages of high densities and easy corrosion, limiting their application in lightweight, wearable electronic equipment. With a high specific surface, outstanding thermal and electrical conductivities, carbon nanomaterials have been studied as lightweight and efficient EMI shielding materials.^4,11–13^ For instance, the carbon nanotube (CNT) sponge with a thickness of 1.8 mm shows a high EMI shielding effectiveness (SE) of 54.8 dB and specific SE (SSE) of 5480 dB cm^3^ g^−1^ in X band.^5^ By adding conductive polymer and magnetic loss materials, the graphene/CNT film with a thickness of 0.6 mm can achieve EMI SE as high as 133.22 dB.^14^

However, in some cases like foldable and flexible electronic devices, the thickness of the shielding films is highly restricted.^15–17^ Moreover, the shielding films are also required to be highly thermally conductive, since electronic components of these devices, such as smart sensors and flexible electrodes,^15,18,19^ produce significant heat emission during operation. If the heat is not effectively dissipated, it will lead to malfunction of these source electronic devices and reduction of their service life.^20,21^ Hence, ultrathin, flexible and thermal conducting films with exceptional EMI SE are demanded.

Graphene has attracted much attention, due to its outstanding thermal conductivity (5000 W m^−1^ K^−1^) and ultrahigh electrical conductivity (∼10^4^ S cm^−1^).^22,23^ Ultrathin graphene films could absorb and reflect EM waves effectively, which was widely studied as efficient shielding materials.^24,25^ Moreover, large-scale production of graphene films could be achieved through reduction of graphene oxide (GO) films.^26,27^ Based on these merits, reduced graphene oxide (rGO) films are promising to be applied as ultrathin thermal conducting films with excellent EMI SE. According to Shen's work, a 8.4 μm-thick rGO film with EMI SE of 20 dB and in-plane thermal conductivity of 1100 W m^−1^ K^−1^ was obtained by thermal annealing of GO film at 2000 °C.^20^ Through improving reduction temperature, higher electrical and thermal conductivities, as well as EMI shielding performance were achieved.^13,21^ By graphitization of GO films at 3000 °C, Xi *et al.* has fabricated the foam-like graphene films, with EMI SE up to 65–105 dB.^28^ The ultrahigh EMI SEs of the foam-like graphene films were attributed to significantly improved electrical conductivity and “expansion enhancement effect” of insulating space layers.

However, the insulating air pores in the graphene films, formed during graphitization of GO films, were detrimental to their electrical and thermal conductivities. Moreover, the porous graphene (PG) films were not mechanically robust enough to meet the requirement of harsh deformations for the wearable and foldable equipments. Cracks were found in the PG films after repeated folding or bending (Fig. S1 and S2†). Compression of the PG films could effectively reduce size of these insulated air pores, thus improving their electrical and thermal conductivity significantly.^27^ The effect of reducing size of these insulated air pores on EMI shielding performance of the graphene films still needs to be studied further, through investigating EMI SEs of both porous graphene films and compressed graphene films.

In this work, large-sized graphene oxide flakes and compression of porous graphene films were employed to prepare ultrathin flexible graphene films with ultrahigh EMI SEs and excellent thermal conductivity. Herein, the porous graphene films were fabricated with the combination of casting-evaporation of GO suspension and thermal annealing at 2600 °C. Due to their better thermal and electrical performances, the porous film made of large-sized graphene flakes (PLG) shows an increase in EMI SE of 7.8 dB, compared to that made of smaller-sized graphene flakes. And four 35.6 μm-thick PLG films were compressed into a 14 μm-thick film (LG-4), with an excellent EMI SE of 73.7 dB and specific SE divided by the thickness (SSE/*t*) of 25 680 dB cm^2^ g^−1^, as well as outstanding thermal conductivity of 803.1 W m^−1^ K^−1^. Moreover, the LG-4 film could endure harsh deformation, with tensile strength of 42.61 MPa and elongation at break of 7.85%, proving to be a promising candidate as thermal conducting materials in wearable and foldable electronic devices.

## Experimental

### Preparation of graphene films

**Fig. 1:**
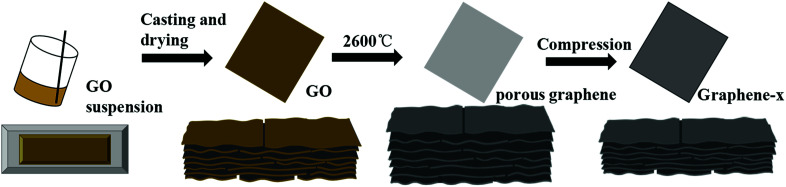
Schematic illustration of preparation process of graphene films (*x* represents the number of PrGO films were compressed together).

Fabrication process of these graphene films is shown in Fig. 1. The abbreviated symbols and thickness of graphene films and graphene oxide films are presented in Table 1.

**Table 1:** Abbreviated symbols for graphene oxide and graphene films (thickness of the porous graphene films is measured from SEM images; thickness of graphene oxide films and compressed graphene films were measured by micrometer)

Samples	Small-size flakes		Medium-size flakes		Large-size flakes	
	Symbols	Thickness	Symbols	Thickness	Symbols	Thickness
Graphene oxide films/(μm)	SGO	15.9 ± 0.5	MGO	16.1 ± 0.4	LGO	16.3 ± 0.4
Porous graphene films (PG)/(μm)	PSG	33.5 ± 0.6	PMG	34.7 ± 0.3	PLG	35.6 ± 0.4
Compressed films with 1 PG layer/(mm)	SG-1	0.0034 ± 0.0003	MG-1	0.0035 ± 0.0004	LG-1	0.0035 ± 0.0003
Compressed films with 2 PG layers/(mm)	SG-2	0.0068 ± 0.0004	MG-2	0.0069 ± 0.0003	LG-2	0.0071 ± 0.0003
Compressed films with 4 PG layers/(mm)	SG-4	0.0134 ± 0.0004	MG-4	0.0138 ± 0.0007	LG-4	0.0140 ± 0.0008

Three kinds of GO/H_2_O dispersions were purchased from Hangzhou Gaoxi Technology Co., Ltd., with GO flakes of different ranges of diameters, *i.e.* 5–8 μm, 20–30 μm and 40–50 μm, noted as SGO, MGO and LGO, respectively. The GO dispersions were diluted to 8.0 mg ml^−1^ with water, and dispersed by mechanical stirring for 4 h, then bar-coated on a PET plate (Fig. S3†). After drying at 35 °C for 24 h, GO films were peeled off from the PET substrate. Areal densities of these GO films were precisely controlled to be around 1.70 mg cm^−2^ by adjusting the concentration of GO suspension and height of scraper at around 2 mm. After graphitization at 2600 °C for 4 h, the reduced graphene films expanded along the thickness direction, forming the porous multi-layer films with shiny metallic luster. And the areal densities decreased to 0.61, 0.69 and 0.71 mg cm^−2^ for PSG, PMG and PLG, respectively. After compression, these porous graphene films turn into ultrathin, flexible graphene films with compacted structure. The compression process is: 50 MPa/10 min + 100 MPa/30 min + 300 MPa/30 min.

## Material characterization

Sizes of the GO flakes, and morphologies of the GO and graphene films were investigated by scanning electron microscope (SEM, Hitachi S4800, FEI, Japan). Raman spectra were performed on a LabRAM HR Raman Spectroscopy, and a laser excitation of 532 nm was employed. The elemental compositions of the samples were investigated by X-ray photoelectron spectroscopy (XPS, PHI 5000C ESCA System, 14.0 kV). X-ray diffraction (XRD) data was collected with a X'Pert Pro (PANalytical) diffractometer using monochromatic Cu Kα1 radiation (*λ* = 1.5406 Å) at 40 kV. Fourier transform infrared (FTIR) spectra were recorded by a Nicolet 10 spectrometer.

Static uni-axial tensile tests of the film samples were conducted on a dynamic mechanical analyzer (D8000 DMA, Perkin Elemer, US). Two ends of the sample, with a length of about 6 mm and width of around 3 mm, were gripped by tension clamps tightly (Fig. S3†). All tensile tests were conducted at 25 °C, in controlled force mode at a loading rate of 0.02 N min^−1^. Sheet resistance of graphene films was measured on a Keithley 2450 Source Meter by the four-probe method, under small currents. Electrical conductivity of all the films can be calculated by the following equation:

1 *σ* = 1/(*R*_s_ × *t*)

where, *σ*, *R*_s_ and *t* are the electrical conductivity, sheet resistance and thickness of the graphene films, respectively. Dimension of graphene films for electrical conductivity testing is 25 mm × 25 mm. Thermal conductivity can be calculated from the equation:

2 *λ* = *α* × *C*_p_ × *ρ*,

where, *λ*, *α*, *C*_p_, and *ρ* are thermal conductivity, thermal diffusivity, specific heat capacity and material density, respectively. Thermal diffusivities of the compressed graphene films were measured by a light flash system (NETZSCH LFA 447) at room temperature. The specific heat capacities were measured from differential scanning calorimeter (DSC, Mettler), and densities of the graphene films were calculated based on sample weight and volume. Diameter of graphene films for thermal conductivity testing is around 25 mm.

*S*-parameters of the samples, including the reflection (*S*_11_ or *S*_22_) and transmission (*S*_12_ or *S*_21_) of a transverse EM wave, were measured by a vector network analyzer (AV3672E, CETE-41) using the wave-guide method in X-band. The standard sample dimensions were 22.86 mm × 10.16 mm, and the tested samples were made by attaching slightly larger graphene films onto a polyurethane (PU) foam substrate. As shown in Fig. S4,† dimension of PU foam is 22.8 mm × 10.2 mm × 3 mm approximately. The EMI shielding effectiveness (SE) was calculated by the following formulas:

3 *R* = |*S*_11_|^2^, *T* = |*S*_12_|^2^, *T* + *R* + *A* = 1

4 SE_ref_ = −10 × log(1 − *R*)

5 SE_abs_ = −10 × log[*T*/(1 − *R*)]

6 SE_total_ = SE_ref_ + SE_abs_

The *A*, *R* and *T* are the absorption, reflection and transmission coefficients, respectively. SE_total_, SE_abs_ and SE_ref_ are the total, absorptive and reflective EMI SE, respectively.

## Results and discussion

### Morphologies of graphene films

**Fig. 2:**
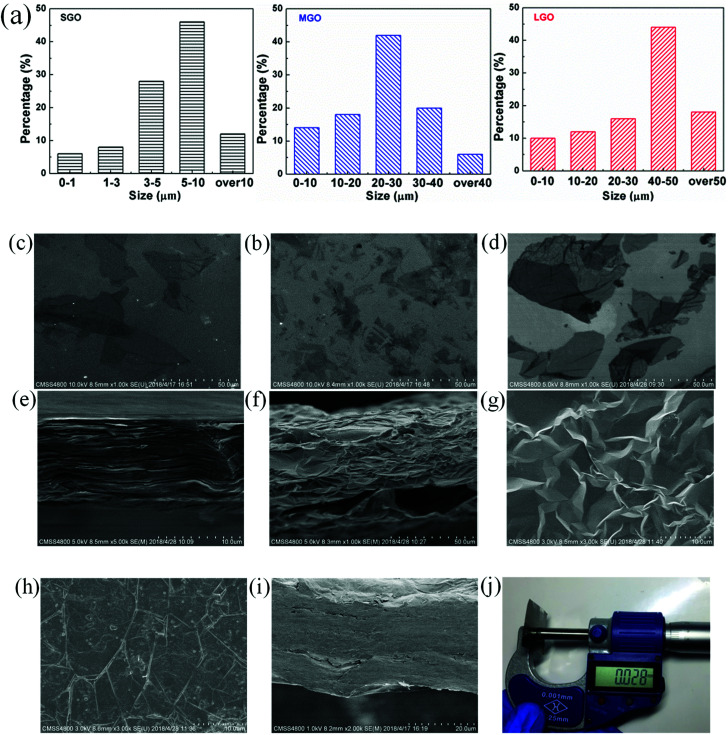
Morphology and structural characterization of GO and graphene films. (a) Corresponding histograms of GO flakes size distribution; SEM images of: (b) small-size flakes, (c) medium-size flakes and (d) large-size flakes; cross-section SEM images of (e) LGO film; (f) PLG film; top views of (g) PLG film, (h) LG-1 film; cross-section SEM images of (i) LG-8 film; (j) picture of thickness measurement.

Sizes of graphene oxide flakes, and morphology of the LGO film were observed by SEM, as shown in Fig. 2a–e. The average sizes of graphene oxide flakes were calculated by measuring the longest end to end point of more than 50 flakes. And the size distribution histograms of SGO, MGO and LGO are shown in Fig. 2a. The average flakes size of LGO is around 50 μm, which is much larger than that of SGO (Fig. 2b–d). As shown in Fig. 2e, the LGO film shows well-order layered structure, formed during evaporation of GO suspension after casting. After graphitization, the expanded graphene film shows porous multi-layer structure, composed of graphene laminates and insulated air pores, which range from several to tens of micrometers (Fig. 2f), due to gas releasing between the graphene laminates. Though the porous graphene films expanded significantly on the thickness direction, the graphene laminates still formed continuous structure, owing to the overlaps among graphene flakes. As shown in Fig. 2f and g, the graphene laminates are closely compacted and non-defective. After compression, the microfolds, which are essential to the foldability and flexibility of graphene films, could be observed on the surface of the LG-1 film (Fig. 2h). Moreover, due to the highly ordered structure, several porous graphene films are easily to be compressed compactly. As shown in Fig. 2i, a 28 μm-thick film was obtained by the compression of 8 PLG films together, which is still flexible and foldable with compacted structure along the cross-section direction. Thickness of the LG-8 film is measured by the micrometer, which is in good agreement with that determined by SEM images (Fig. 2i and j).

### Characterization of the modified graphene films

#### XPS, XRD, Raman and FTIR spectra of as-prepared graphene oxide and graphene films

**Fig. 3:**
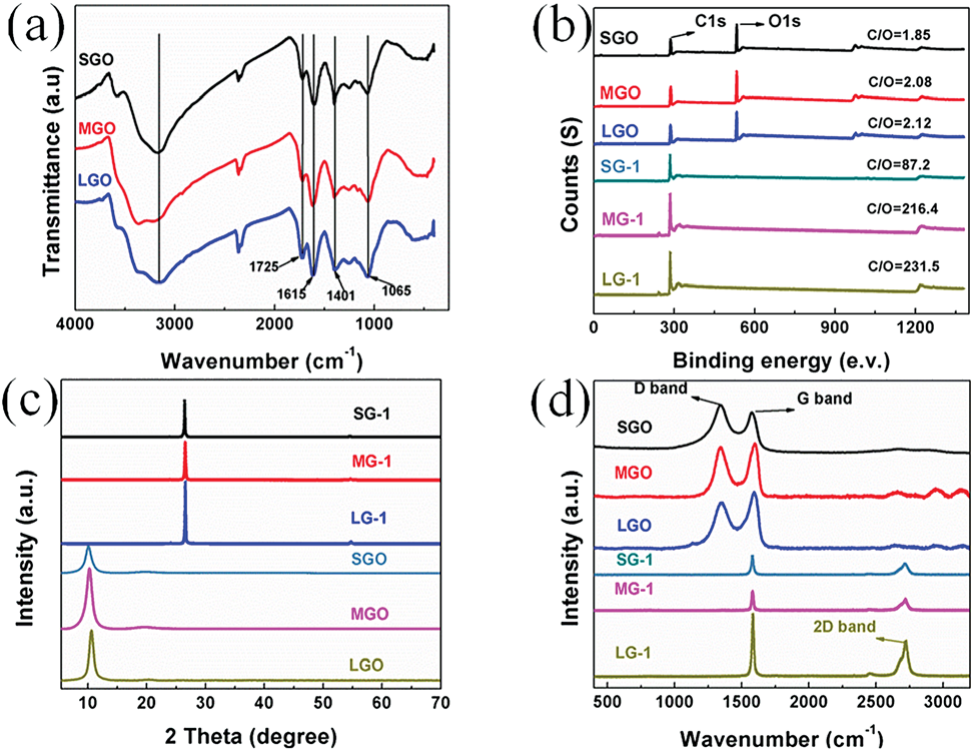
(a) FT-IR spectra of SGO, MGO and LGO; (b) XPS survey spectra, (c) XRD spectra, and (d) Raman spectra of the GO films and graphene films.

The chemical compositions and molecular structures of GO and graphene films with varied flakes sizes are systematically characterized by FT-IR, XPS, XRD and Raman spectroscopy. As shown in Fig. 3a, FT-IR spectra of all GO samples exhibit peaks of functional groups, corresponding to hydroxyl stretching vibrations (∼3430 cm^−1^), carboxyl stretching vibrations (∼1725 cm^−1^), aromatic carbon bonds (∼1615 cm^−1^), and epoxy and alkoxy bonds (∼1065 cm^−1^). From Fig. 3a, it can be drawn that the intensity ratios of aromatic carbon to carboxyl bonds increase, when sizes of graphene flakes increase, which is further confirmed by XPS tests.

The XPS spectra of all GO and porous graphene films is shown in Fig. 3b. Generally, the larger the size of the graphene flakes, the higher the atomic ratios of carbon to oxygen (C/O). In this work, C/O ratio of the SGO film is 1.85, which increases to 2.12 for the LGO film, indicating that the LGO film has relatively less oxygenated groups. After graphitization, as seen in the survey spectrum, oxygen can be barely observed for all reduced graphene films, owing to the elimination of most oxygenated groups. C/O ratio of the SG-1 film is 87.2, which is lower than that of the LG-1, indicating a few remaining defects and oxygenated groups for the SG-1 film.

Fig. 3c shows the XRD patterns of as-prepared GO films and compressed graphene films. The characteristic diffraction peaks of GO films appear at 10.02°, 10.31° and 10.60° for the SGO, MGO and LGO films, corresponding to the *d*-spacing around 8.8, 8.5 and 8.3 Å, respectively. After graphitization, the 2*θ* of graphene films were shifted to 26.42°, 26.52° and 26.58° for SG-1, MG-1 and LG-1, respectively. The shift of 2*θ* suggests that the GO films were well reduced during thermal treatment. Moreover, *d*-spacing of these compressed graphene films are slightly greater than that of the natural graphite, attributed to residual structural defects. Due to higher degree of compactness and more ordered structure during assembling of graphene flakes, *d*-spacing of the LG-1 film (3.352 Å) is a bit smaller than that of the SG-1 film (3.368 Å).

Raman spectroscopy of the GO films and graphene films are shown in Fig. 3d, from which two noticeable peaks could be observed at about 1354 cm^−1^ and 1592 cm^−1^ for GO films, corresponding to D band and G band. The peak intensity ratio of D band to G band (*I*_D_/*I*_G_) is used to characterize structural defects and sp^2^ hybridized domains. The SGO film possesses the *I*_D_/*I*_G_ of 1.74, which is higher than that of the MGO (1.65) and the LGO (1.53). The results demonstrate that larger-sized graphene flakes has less oxygenated groups and fewer defects, which might lead to higher electrical conductivity and better EMI shielding performance. After graphitization, a new peak appears at 2718 cm^−1^ for the compressed graphene films, corresponding to the 2D peak, which indicates the formation of graphite structure during thermal treatment.

#### EMI sheilding performance of graphene films

**Fig. 4:**
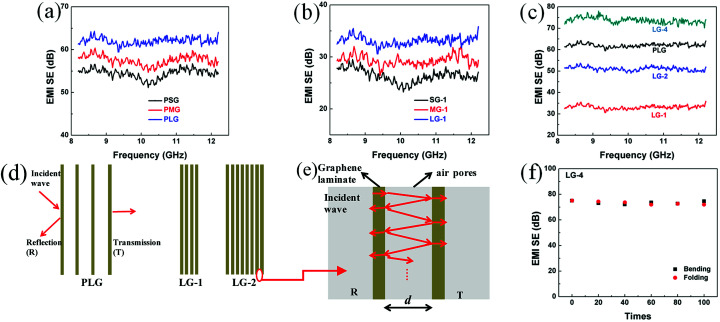
The EMI SEs of (a) the PG films, (b) the graphene films compressed with one PG film, (c) the graphene films made of large-sized flakes; (d) an ideal multi-layer model for PLG and compressed graphene films composed of graphene laminates and insulated air pores; (e) schematic representation of a local multi-layer model composed of two graphene laminates and insulated pores between two graphene laminates; (f) EMI SEs of the LG-4 film after bending and folding.

EMI shielding performance of the PG films and compressed graphene films made of varied flakes sizes were investigated. As shown in Fig. 4a and b, both the PLG and LG-1 films show higher EMI SEs than the films made of smaller-sized flakes. For instance, EMI SE of the PLG film is 7.8 dB higher than that of the PSG film. After compression, the SG-1 film possesses EMI SE of 26.4 dB, which increases to 29.3 dB for the MG-1 film and 33.1 dB for the LG-1 film, respectively. Interestingly, EMI SE of the PLG film decreased significantly from 62.0 dB to 33.1 dB after compression, which is mainly attributed to compression of insulated air pores in the porous multi-layer structure. As shown in Fig. 2f, the PLG film expanded along the thickness direction, resulting in porous multi-layer structure composed of graphene laminates, insulated air pores between graphene laminates and interfaces of graphene laminates and air pores.

A multi-layer model and electromagnetic theories are applied to study the effect of air pores on EMI shielding performance of graphene films.^28^ As shown in Fig. 4d, it is assumed that graphene films are composed of homogenous graphene laminates and insulated pores. And the average thickness of air pores is *d*, which decreased significantly for LG-1 and LG-2 after compression. As shown in Fig. 4e, A local model was extracted from Fig. 4d, which is composed of two graphene laminates, insulated pores and two interfaces. At the interface of graphene laminate and air pores, a portion of waves would reflect and the others will transmit through the interface. Then the reflected waves would be reflected to another interface and experience reflection and transmission again. Therefore, the incident waves will be reflected and transmitted infinitely between two graphene laminates. The effective transmission coefficient (*τ*_eff_) is defined as the ratio of total transmitted EM waves to incident waves, which could be calculated by the eqn (7):^29^7
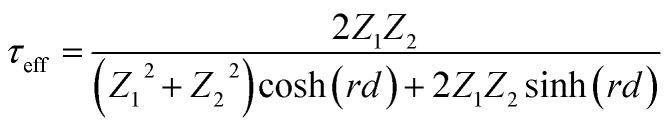
where, *Z*_1_ and *Z*_2_ are wave impedance of free space and graphene materials, *r* is propagation constant of free space, and *d* is average distance between adjacent graphene laminates. On the condition that *d* ranges from 0 to 100 μm and *f* is from 8.2 to12.2 GHz, *τ*_eff_ is obviously lower than 1. Therefore, the air pores contribute significantly to shielding performance. With smaller *d*, compressed graphene films show much lower EMI SE, compared with porous graphene films. On the condition that *d* = *λ*/2 and *r* = *j* × 2 × π/*λ* (*λ* is wavelength), *τ*_eff_ is calculated as −1, indicating that destructive interference of waves might happen. Hence, the excellent EMI shielding performance of the porous graphene films is probably because of the interference among the component waves, which is not related to the number of graphene laminates in the multi-layer structure, but related to the size of air pores. For the PLG films, although insulated air pores could not consume waves directly, these air pores produce multiple interfaces of graphene laminates and air pores, contributing to dispersing extra waves due to the wave interference.

Though the PG films possess higher EMI SEs, the insulated air pores would significantly decrease their electrical and thermal conductivities. Hence, compression of PG films is necessary to obtain ultrathin, flexible thermal conducting films. To improve the relatively low EMI SEs of compressed graphene films, adding the thickness of shielding materials is simple and efficient. Four layers of PLG films were compressed together to obtain the 14 μm-thick LG-4 film, leading to ultrahigh EMI SE of 73.7 dB (Fig. 4c). And, EMI SE of the ultrathin LG-4 film, with smaller air pores, surpasses that of the FLG film, which is mainly attributed to increased graphene laminates and increased interfaces between graphene laminates and pores (Fig. 4d). Besides, the 14 μm-thick LG-4 film is still mechanically robust, which can be even folded into complicated shape without any breakage or cracks. After repeated bending (bending speed of ∼0.037 Hz and bending radius from ∞ to 0 mm, as shown in Fig. S5†) or folding for 100 times, EMI SEs of the LG-4 film are almost unchanged (Fig. 4f), indicating that these harsh deformations have little influence on its EMI shielding performance.

**Fig. 5:**
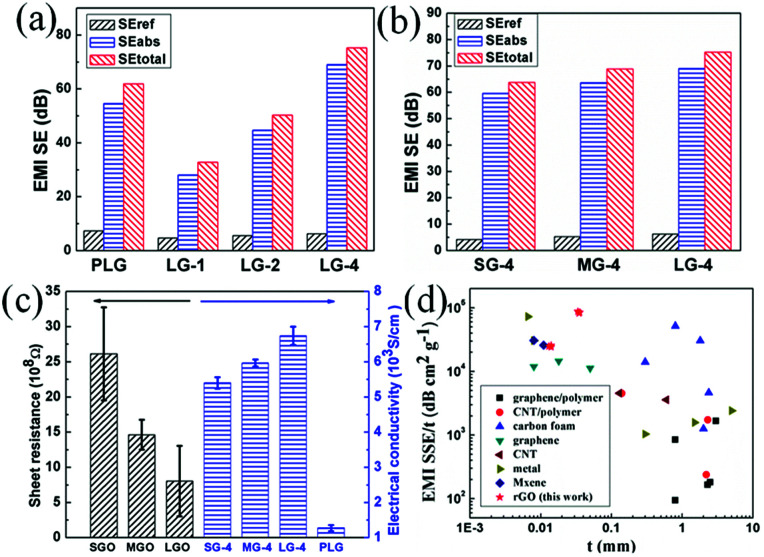
SE_ref_, SE_abs_ and SE_total_ at 10 GHz of (a) the PLG, LG-1, LG-2, LG-4 films, (b) the SG-4, MG-4, LG-4 films; (c) electrical conductivity of GO films and graphene films; (d) EMI SSE/*t versus* thickness of the graphene films, in comparison to different materials in the previous literature, and *t* represents thickness.

To further study the enhanced mechanism of EMI shielding performance of graphene films, the SE_total_, SE_ref_ and SE_abs_ at 10 GHz of the graphene films made of large-sized flakes and the SG-4, MG-4 films were calculated (Fig. 5a–b). As shown in Fig. 5a, SE_ref_ of the PLG film is the highest among these films, indicating that the PLG film reflects more EM radiation than other films. After compression of the PLG film, SE_total_ of LG-1 film is significantly reduced, mainly attributed the remarkable decrease of SE_abs_ and slight decrease of SE_ref_. According to eqn (7), the decreasing distance between graphene laminates after compression results in decrease of shielding performance for the PLG film. Comparing with the PLG film, the LG-4 film possesses better EMI SE, because of higher SE_abs_, which is proportional to increased thickness of graphene laminates. Obviously, SE_abs_ of the LG-4 film is several times higher than its reflection loss, which indicates that the LG-4 film is a absorption-dominant EMI shielding material, consistent with the reported results of carbon-based materials.^30–32^ Moreover, as shown in Fig. S6,† the absorption and reflection coefficients of the LG-4 film are around 0.160 and 0.839, respectively. And the transmission coefficient is close to zero, meaning that most of electromagnetic waves are blocked and absorbed by the LG-4 film. As displayed in Fig. 5b, reflection loss of these graphene films compressed with four PG films are nearly the same. Therefore, the higher EMI SE for the LG-4 film results from the increased SE_abs_ loss and smaller portion of EM radiation entering into shielding materials, which is mainly attributed to higher electrical conductivity of the large-sized graphene films.

The electrical conductivity of GO and graphene films were investigated, as shown in Fig. 5c. As the size of graphene flakes increases, the sheet resistance decreases from 26.1 × 10^8^ (SGO) to 8.1 × 10^8^ Ω (LGO), due to less defects and functional groups. After graphitization, electrical conductivity of the porous graphene films show a remarkable increase, due to defects restoration during thermal treatment. The electrical conductivity of the PSG film is measured as 1170 S cm^−1^, which increases to 1270 S cm^−1^ for the PLG film. After compression, electrical conductivity of the LG-4 film increases several times, up to 6740 S cm^−1^. Compared with graphene films made of smaller-size flakes, large-sized graphene films possess higher electrical conductivity, leading to better EMI shielding performance. Moreover, a small increase of electrical conductivity would lead to a significant decrease in the skin depth of a shield.^33,34^ And the decrease in the skin depth for the LG-4 film results in the significant enhancement of SE_abs_. The skin depth (*δ*) is calculated according to the following equation:

8 *δ* = (π*σfμ*)^−1/2^

where, *σ* is the electrical conductivity, *f* is the frequency and *μ* is the magnetic permeability. On the conditions that the electrical conductivity of LG-4 is 6960 S cm^−1^, *μ* keeps constant to be *μ*_0_, and *f* is at 10 GHz, the calculated value of *δ* is 6.0 μm based on eqn (8).

Although the thickness of LG-1 film (0.0035 mm) is thinner than the calculated *δ*, EMI SE of LG-1 still reaches to 33.1 dB, due to the consumption of more EM energy by the multi-layer structure of shielding materials. With nearly the same thickness, EMI SE of the LG-4 film is higher than that of the SG-4 and MG-4 films, due to smaller *σ* for the LG-4 film. Complex permittivity (real part ε′ and imaginary part *ε*′′) and loss tangent (tan *δ*) are analyzed to further study shielding mechanism, as presented in Fig. S9.†

To evaluate lightweight EMI shielding materials, like foldable or wearable electronic devices, the specific shielding effectiveness divided by thickness (SSE/*t*) has been widely used, when taking density and weight into account. Notably, the-14 μm-thick LG-4 film possesses EMI SE of 73.7 dB, which is higher than the 10 μm-thick copper foil (70 dB, Table S6†). And SSE/*t* of the LG-4 film is nearly 3 times higher than that of the copper foil. In our work, SSE/*t* of the LG-4 film reaches to 25 680 dB cm^2^ g^−1^, superior to most of the reported works (Fig. 5d).^1,4,5,11,13,19,20,28,31,32,35–51^ This finding is noteworthy because the LG-4 film would satisfy several commercial requirements for an EMI shielding material, for example ultrahigh EMI SE (73.7 dB), low density (2.05 g cm^−3^), ultrathin thickness (0.014 mm), anti-corrosion, high flexibility and easy fabrication.

#### Thermal conductivity analysis

**Fig. 6:**
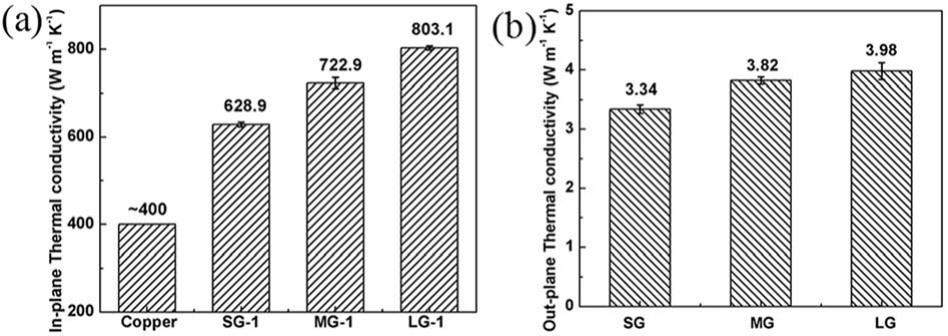
(a) In-plane thermal conductivity and (b) out-plane thermal conductivity of compressed graphene films.

The excellent electrical conductivity of compressed graphene films implied that these films would show high thermal conducting property. A light flash system was adopted to determine in-plane thermal conductivity of the graphene films. As shown in Fig. 6a, the SG-1 film possessed an excellent thermal conductivity of 628.9 W m^−1^ K^−1^, which is much higher than copper (one of the best heat conductors, ∼400 W m^−1^ K^−1^).^20^ Compared with that of the SG-1 film, a 27.9% improvement of thermal conductivity was observed for the LG-1 film, which is attributed to the reduced phonon-boundary scattering resulted from more compacted and ordered structure of graphene films made of large-sized graphene flakes with fewer defects. With the size of graphene flakes increasing, acoustic phonons with longer wavelengths are available for heat transfer, leading to higher thermal conductivity.^52,53^ However, the out-plane thermal conductivities of graphene films are quite low, compared with their in-plane thermal conductivities (Fig. 6b). Due to fewer defects of graphene sheets, the LG film possesses higher out-plane thermal conductivity than the SG and MG films.

#### Mechanical property analysis

**Fig. 7:**
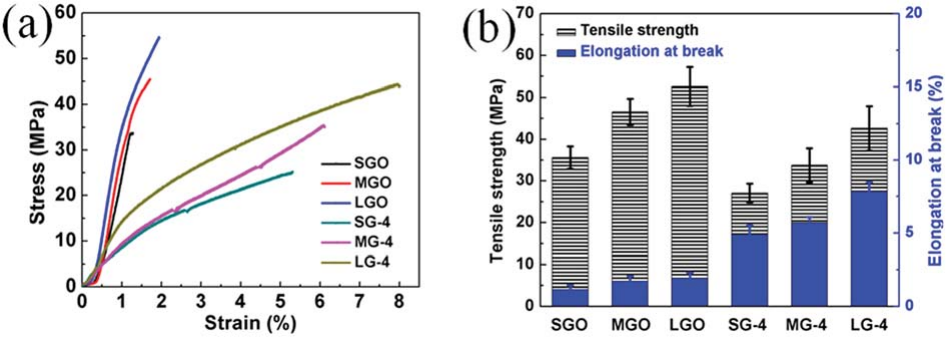
Tensile properties of GO and compressed graphene films: (a) stress–strain curves and (b) tensile strength and elongation at break.

The ultrathin graphene films in this research have not only superior electrical and thermal properties, also excellent mechanical performance. Representative stress–strain curves of GO and graphene films are shown in Fig. 7a. According to these curves, when size of graphene flakes increases, both tensile strength (*σ*) and elongation at break (*ε*_b_) are improved for these GO and compressed graphene films. For example, the *σ* of the SGO film is 35.6 MPa, and the *ε*_b_ is 1.15%. Those of the LGO film are significantly improved to 52.6 MPa and 1.93%, when the average flakes sizes increased from 5–8 μm to 40–50 μm. This size effects induced enhancement is due to less defects and stronger π–π interaction between graphene flakes for the LGO film.^9^

After graphitization, a slight decrease of *σ* is observed for all graphene samples, while *ε*_b_ of graphene films is dramatically increased. As shown in Fig. 7b, the LG-4 film shows a high *ε*_b_ of 7.85%, which is in good agreement with its high flexibility and foldability. And the SEM images (Fig. 2h–i) reveal that the excellent mechanical property of graphene films should be attributed to microfolds of the compressed graphene films, which enable graphene films to recover the original structure after repeated deformation without any crack or breakage.

The above results demonstrate that the LG-4 film is promising to be used as thermal conducting films in wearable or foldable electronic devices, which require high EMI SE, low density, ultrathin thickness, excellent thermal conductivity and high flexibility.

## Conclusion

In conclusion, ultrathin and flexible graphene films were fabricated with large-sized graphene flakes, which displayed outstanding EMI shielding performance, thermal conductivity and mechanical properties. The 14 μm-thick LG-4 film possesses EMI SE of 73.7 dB and the SSE/*t* of 25 680 dB cm^2^ g^−1^, which is one of the highest among the reported values. The superior EMI shielding performance is attributed to less defects, excellent electrical conductivity and multi-layer structure of the LG-4 film. The LG-4 film also shows high thermal conductivity of 803.1 W K^−1^ m^−1^, and excellent mechanical flexibility with a elongation at break of 7.85%. The above results indicate that the LG-4 film shows great potential as excellent thermal conducting films applied in wearable or foldable electronic devices, which require lightweight, high flexibility, thermal conductivity, and efficient EMI shielding performance.

## Conflicts of interest

There are no conflicts to declare.

## Supplementary Material

RA-009-C8RA09376H-s001
