# Supplementary material for: Ultrathin flexible graphene films with high thermal conductivity and excellent EMI shielding performance using large-sized graphene oxide flakes
Source: RSC Adv. 2019 Jan 11;9(3):1419–27. doi: 10.1039/c8ra09376h (PMC9059650; doi:10.1039/c8ra09376h)
Supplement: RA-009-C8RA09376H-s001 [file RA-009-C8RA09376H-s001.pdf]

Electronic Supplementary Material (ESI) for RSC Advances.
This journal is © The Royal Society of Chemistry 2018

Supplementary Information

Ultrathin flexible graphene films with high thermal conductivity and excellent EMI shielding performance using large-sized graphene oxide flakes

Shaofeng Lin, Su Ju, Jianwei Zhang*, Gang Shi, Yonglyu He and Dazhi Jiang*

Corresponding author. Tel: 0086-731-84507226

Email: jwzhang.nudt@gmail.com, jiangdz@nudt.edu.cn

Department of Materials Science and Engineering, National University of Defense Technology; Changsha, Hunan, 410073, People Republic of China

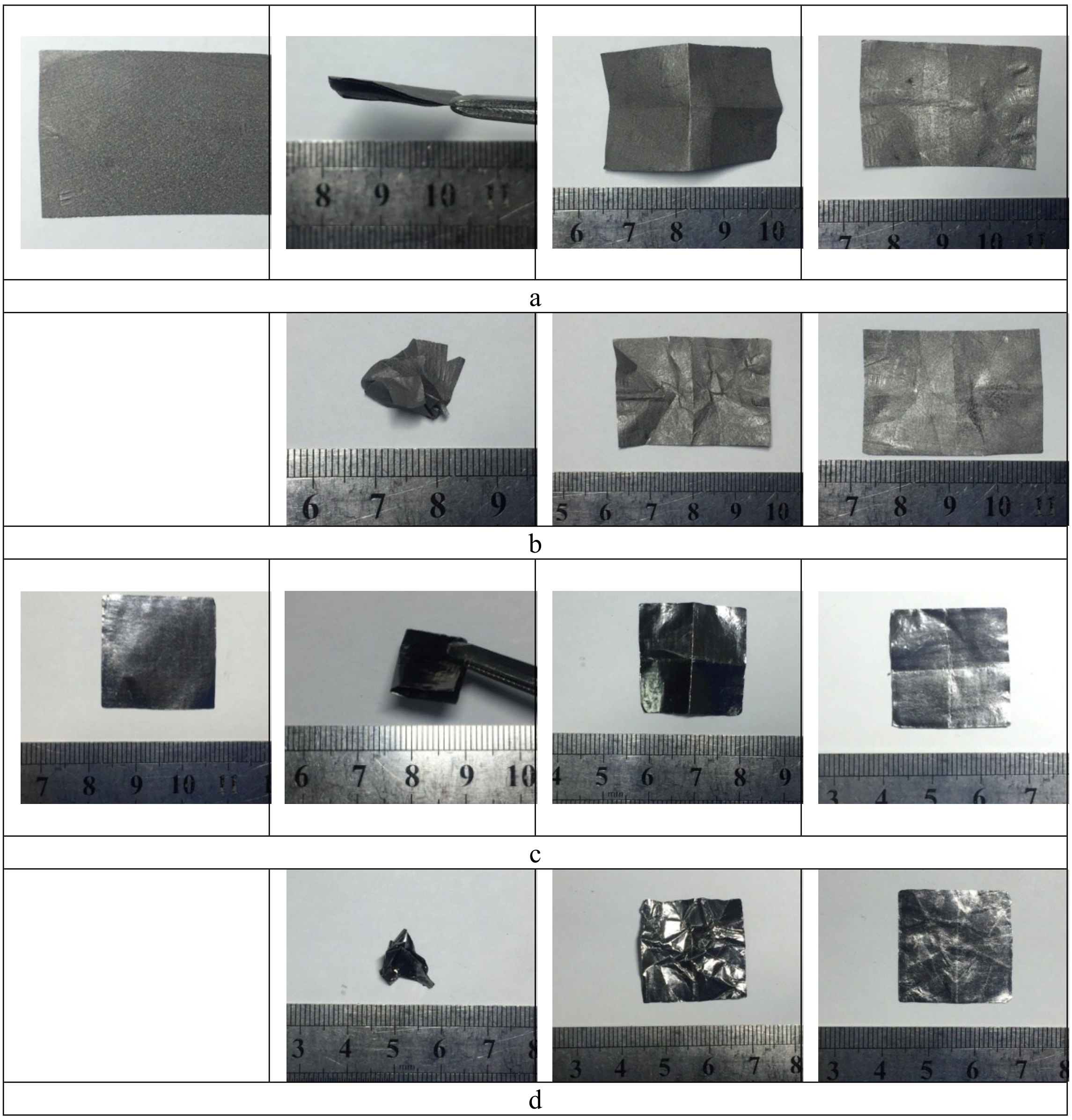

Figure S1 Images of (a) folding, then unfolding, and (b) crumpling, then releasing of the PLG film; (c) folding , then unfolding, and (d) crumpling, then releasing of the LG-4 film.

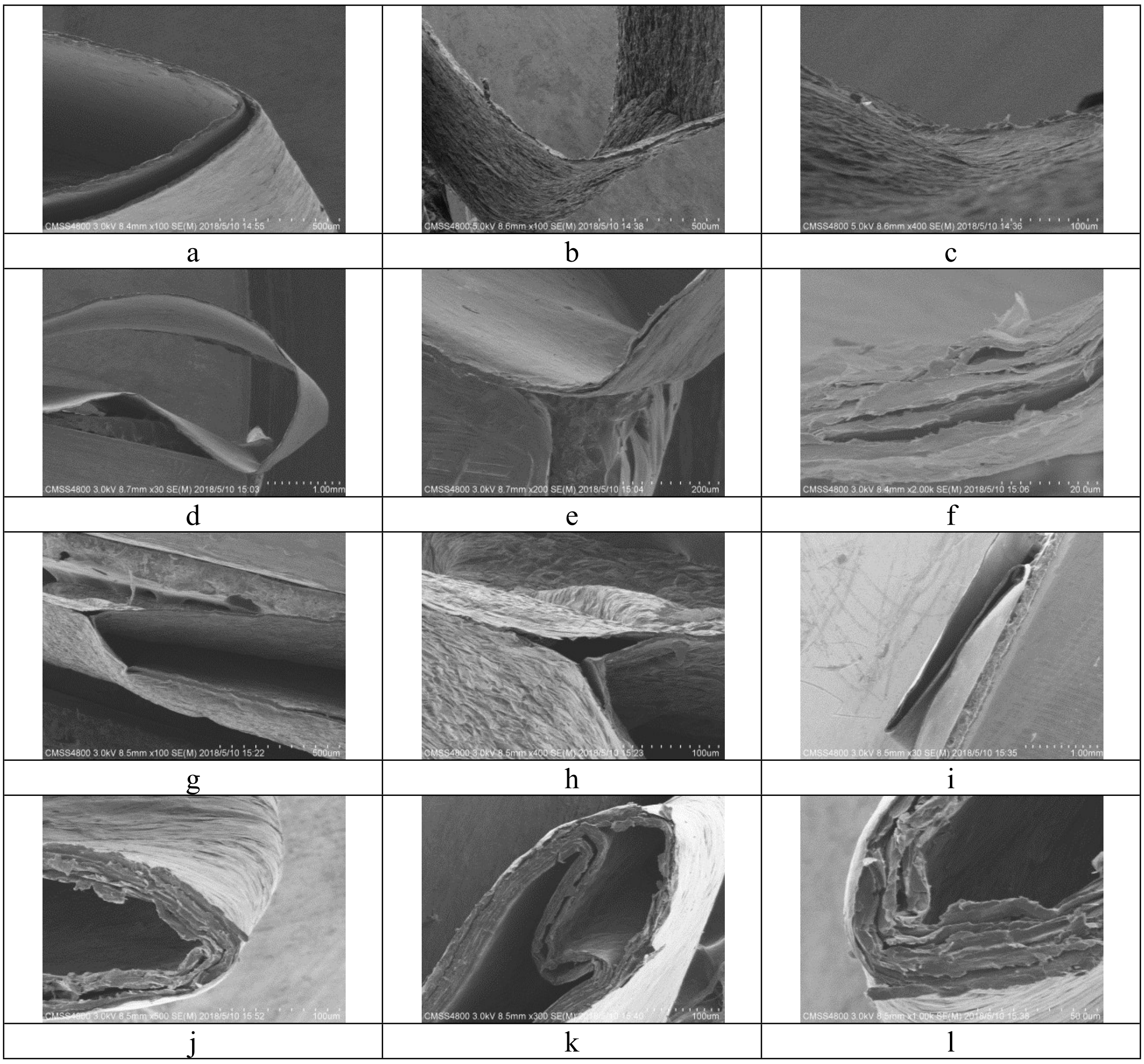

Figure S2 SEM images of (a) double folding, (b-f) bending of PLG films; (g-h) cracks of the PLG film after repeated folding; (i-k) folding, (l) bending of the LG-4 film.

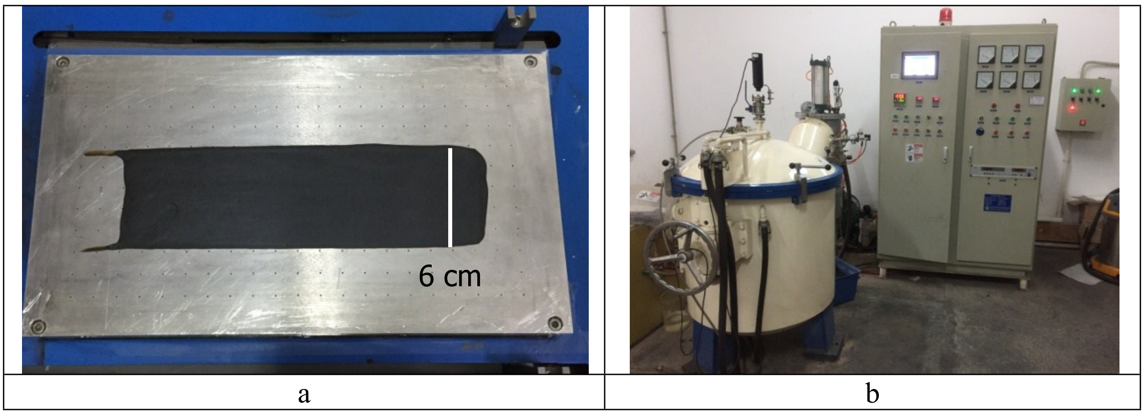

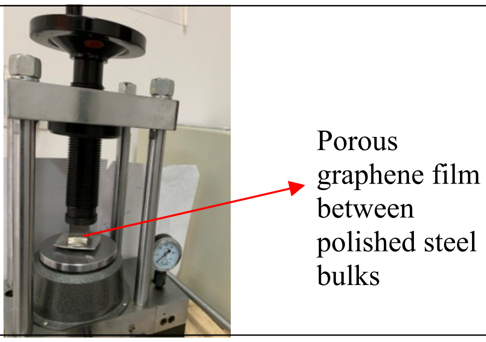

c

Figure S3 Fabrication of graphene films. (a) casting and drying of GO suspension; (b) the annealing furnace; (c) compression of porous graphene films.

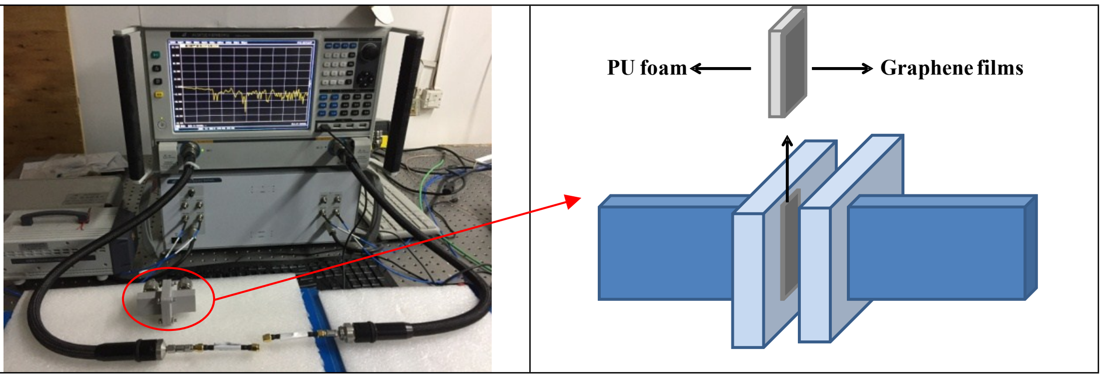

Figure S4 EMI shielding performance testing equipment.

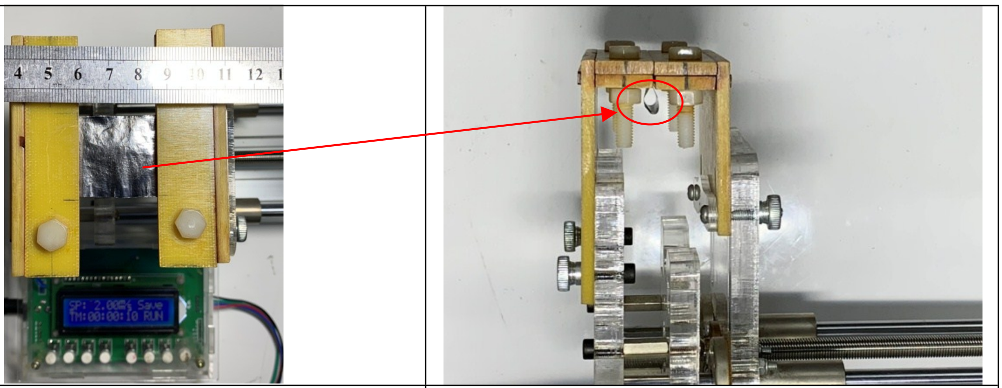

(a) (b)

Figure S5 Bending testing of compressed graphene films: (a) before bending; (b) after bending.

As shown in Figure S5, the moving speed is 2 mm·s^{-1} and free length of graphene films is around 27 mm. The bending radius is from ∞ to 0 mm and the bending speed is around 0.037 Hz. After repeated bending, no cracks or breakages were formed for the compressed graphene films.

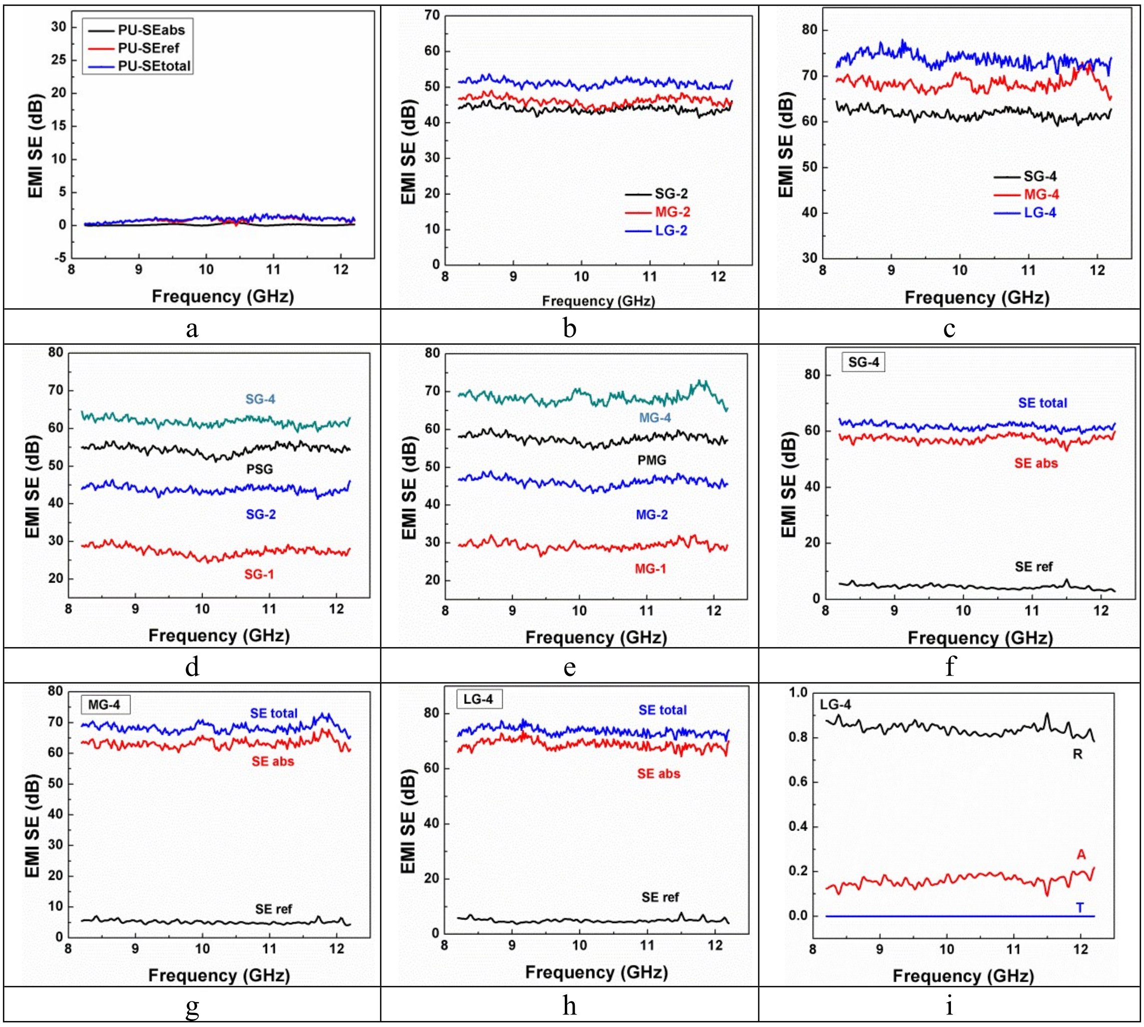

Figure S6 EMI SE of (a) PU substrate, (b) graphene films compressed with two porous graphene films, (c) graphene films compressed with four porous graphene films, (d) small-size graphene films, (e) medium-size graphene films; SE_{ref}, SE_{abs} and SE_{total} of (f) the SG-4 film, (g) the MG-4 film, and (h) the LG-4 film; (i) A and R coefficients of the LG-4 film.

Table S1 The electrical conductivity of graphene films and the copper foil

Samples	Electrical conductivity 10^4 (S·cm^{-1})	STDEV 10^4 (S·cm^{-1})	Samples	Electrical conductivity 10^4 (S·cm^{-1})	STDEV 10^4 (S·cm^{-1})
PSG	0.117	0.011	SG-2	0.540	0.016
PMG	0.121	0.009	MG-2	0.597	0.010
PLG	0.127	0.016	LG-2	0.674	0.026
SG-1	0.523	0.009	SG-4	0.541	0.012
MG-1	0.601	0.017	MG-4	0.585	0.017
LG-1	0.645	0.023	LG-4	0.696	0.016
Cu	50.2	0.4			

Table S2 Sheet resistance of GO films

Samples	Sheet resistance (10^8 Ω)	STDEV (10^8 Ω)	Areal density($mg\cdot cm^{-2}$)
SGO	26.13	3.62	1.72
MGO	14.65	2.11	1.69
LGO	8.07	5.01	1.68

Table S3 The in-plane thermal conductivity of compressed graphene films

Samples	Diffusivity ($mm^2\cdot s^{-1}$)	STDEV ($mm^2\cdot s^{-1}$)	Specific heat capacity ($J\cdot g^{-1}\cdot K^{-1}$)	Density ($g\cdot cm^{-3}$)	Thermal conductivity ($W\cdot K^{-1}\cdot m^{-1}$)
SG-1	465.06	3.99	0.708	1.91	628.9
MG-1	475.31	8.34	0.753	2.02	722.9
LG-1	501.63	4.5	0.781	2.05	803.1

Table S4 The out-plane thermal conductivity of compressed graphene films

Samples	Diffusivity ($mm^2\cdot s^{-1}$)	Thickness (μm)	Thermal conductivity ($W\cdot K^{-1}\cdot m^{-1}$)
SG	2.47±0.05	45.4	3.34±0.07
MG	2.51±0.04	46.1	3.82±0.06
LG	2.53±0.09	47.2	3.98±0.14

Table S5 The tensile tests of graphene oxide films and compressed graphene films

Sample	Strength (MPa)	STDEV (MPa)	Elongation at break (%)	STDEV (%)
SGO	35.60	2.67	1.15	0.61
MGO	46.54	3.13	1.73	0.45
LGO	52.62	4.61	1.93	0.52
SG-4	27.08	2.27	4.91	0.61
MG-4	33.70	4.12	5.70	0.40
LG-4	42.61	5.29	7.85	0.62

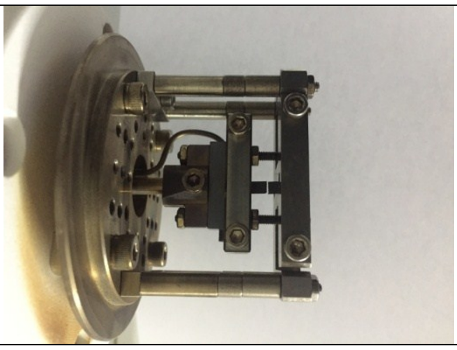

Figure S7 The tensile tests of graphene oxide films and compressed graphene films

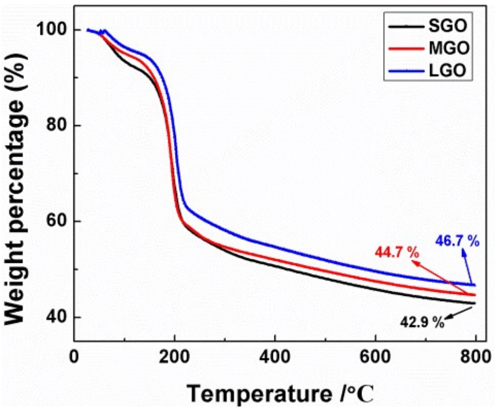

Figure S8 TGA spectrum of the SGO, MGO and LGO films.

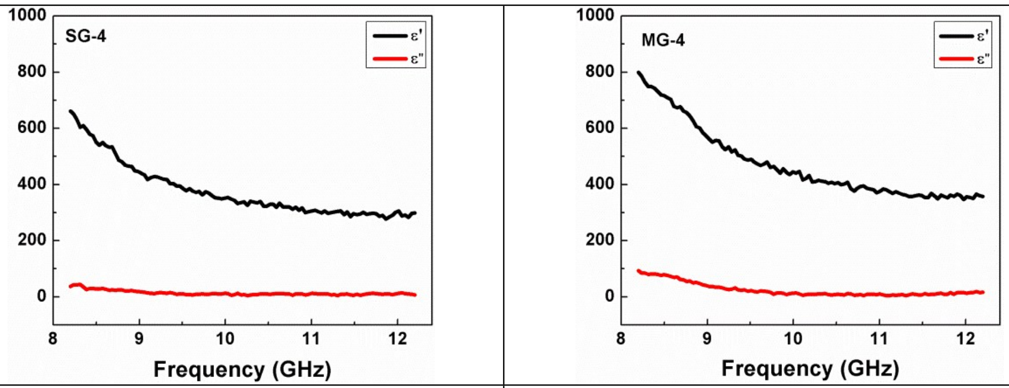

(a)	(b)

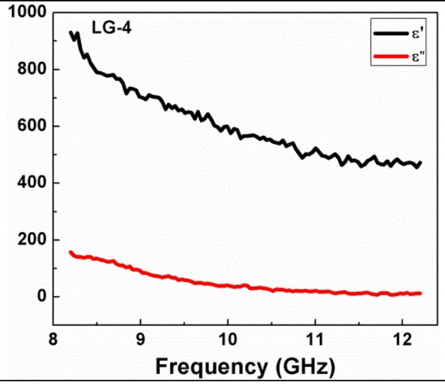

(c)

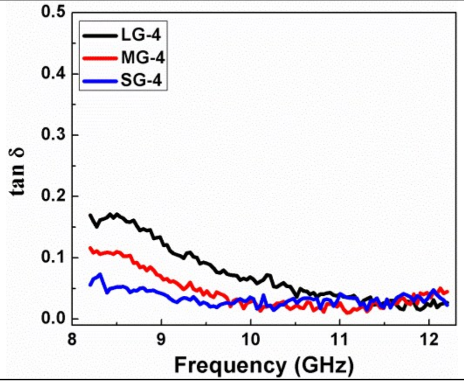

(d)

Figure S9 Complex permittivity and loss tangent (tan δ) of compressed graphene film. The real part ε' and imaginary part ε'' of complex permittivity for (a) SG-4, (b) MG-4 and (c) LG-4 films; tan δ of these graphene films.

Dielectric constant of the samples is tested by the wave-guide method in the X band (8.2-12.2 GHz). Graphene films are attached on the PU foam, with dimensions of 22.8 mm × 10.2 mm × 3 mm. PU is almost transparent to the waves. As shown in Figure S9, dielectric properties of compressed graphene films are characterized. The real part ε' and imaginary part ε'' of complex permittivity of graphene films declined when frequency increased, due to relaxation effect. Because of better electrical conductivity and fewer defects, delocalized π electron of the LG-4 films are more likely to form electric dipole and be polarized, leading to higher dielectric constants than the SG-4 and MG-4 films. Moreover, tan δ of the LG-4 films is also high than other two films, as shown in Figure S9 (d).

Table S6 Specific EMI shielding performance optimized with thickness of varied shielding materials

Materials type	Frequency (GHz)	Density (g cm^{-3})	t (mm)	SE (dB)	SSE/t (dB·cm^2·g^{-1})	Ref
Graphene/PDMS	8-12	0.06	3	30	1667	[1]
Graphene/PEI	8-12	0.29	2.3	11	164.9	[2]
Graphene/PI	8-12	0.28	0.8	21	93.75	[3]
rGO/PANI/PEI	8-12	0.4	2.5	18.2	180	[4]
rGO/PEDOT	8-12		0.8	70	841	[5]
CNT/WPU	8.2-12.4	0.125	2.3	50	1739	[6]
CNT/phenolic	12-18	0.51	0.14	32.4	4537.8	[7]
CNT/pp	8.2-12.4	~0.92	2.2	48.3	238.6	[8]
CNT sponge	2-18		2.38	22	4622	[9]
CNT sponges	8.2-12.4	0.01	1.8	54.8	30444	[10]
Carbon foam	8.2-12.4	0.06	0.3	25.2	14000	[11]
Carbon foam	0.75-1.12		2	40	1250	[12]
Carbon foam	8-12	0.0058	0.8	24	51700	[13]

Graphene foam	2-18		0.120		100000	[14]
Graphene film	8-12	1.07	0.05	60	11214	[15]
Graphene film	8-12	2.1	0.008	20	11904	[16]
Graphene film	0-18		0.018	55	14550	[17]
Expanded graphite	8.2-12.4	1.75	43	52.6	6990	[18]
Graphene/CNT	2-18	1.45	15	57.6	26480	[19]
MWCNT/SWCNT film	8.2-12.4	0.011	0.13	65	4545.5	[20]
CNT	8.2-12.4	0.26	0.6	56	3589	[21]
Graphene film/Fe_3O_4	8.2-12.4	0.78	0.3	21-24	1033	[22]
CuNi-CNT	8-12		1.5	54.6	1580	[23]
Ag nanowires/PI	8-12		5	35	2416	[24]
Silver	8.2-12.4	0.0033	0.0002	43	645600	[25]
silver/MWCNT	0.5-1.0	0.51	0.0066	24.5	72700	[26]
Al	8.2-12.4	2.705	0.008	66	30555	[27]
Cu		8.974	0.010	70	7812	
$Ti_3C_2T_x$		2.394	0.011	68	25863	
$Ti_3C_2T_x$		2.317	0.008	57	30830	
PSG	8.2-12.2	0.0205	0.0335	54.2	88850	This work
PMG		0.0240	0.0347	57.6	84710	
PLG		0.0253	0.0356	62.0	87320	
SG-1		0.1969	0.0034	26.4	39440	
MG-1		0.2018	0.0035	29.3	41490	
LG-1		0.2049	0.0035	33.1	46160	
SG-2		0.2009	0.0068	43.8	32060	
MG-2		0.2052	0.0069	46.2	32630	
LG-2		0.2060	0.0071	51.1	34940	
SG-4		0.1962	0.0134	61.7	23470	
MG-4		0.2045	0.0138	68.4	24240	
LG-4		0.2050	0.0140	73.7	25680	

t represents for thickness; The thickness is measured by micrometer.

[1] Chen Z, Xu C, Ma C, Ren W, Cheng HM. Lightweight and flexible graphene foam composites for high-performance electromagnetic interference shielding. Advanced materials. 2013;25(9):1296-300.

[2] Ling J, Zhai W, Feng W, Shen B, Zhang J, Zheng W. Facile preparation of lightweight microcellular polyetherimide/graphene composite foams for electromagnetic interference shielding. ACS applied materials & interfaces. 2013;5(7):2677-84.

[3] Li Y, Pei X, Shen B, Zhai W, Zhang L, Zheng W. Polyimide/graphene composite foam sheets with ultrahigh thermostability for electromagnetic interference shielding. RSC Advances. 2015;5(31):24342-51.

[4] Shen B, Zhai W, Tao M, Ling J, Zheng W. Lightweight, multifunctional polyetherimide/graphene@Fe3O4 composite foams for shielding of electromagnetic pollution. ACS applied materials & interfaces. 2013;5(21):11383-91.

[5] Agnihotri N, Chakrabarti K, De A. Highly efficient electromagnetic interference shielding using graphite nanoplatelet/poly(3,4- ethylenedioxythiophene) - poly(styrenesulfonate) composites with enhanced thermal conductivity. RSC Advances. 2015;2015(5):43765-71.

[6] Zeng Z, Jin H, Chen M, Li W, Zhou L, Zhang Z. Lightweight and Anisotropic Porous MWCNT/WPU Composites for Ultrahigh Performance Electromagnetic Interference Shielding. Advanced Functional Materials. 2016;26(2):303-10.

[7] Teotia S, Singh BP, Elizabeth I, Singh VN, Ravikumar R, Singh AP, et al. Multifunctional, robust, light-weight, free-standing MWCNT/phenolic composite paper as anodes for lithium ion batteries and EMI shielding material. RSC Adv. 2014;4(63):33168-74.

[8] Wu H-Y, Jia L-C, Yan D-X, Gao J-f, Zhang X-P, Ren P-G, et al. Simultaneously improved electromagnetic interference shielding and mechanical performance of segregated carbon nanotube/polypropylene composite via solid phase molding. Composites Science and Technology. 2018;156:87-94.

[9] Crespo M, González M, Elías AL, Pulickal Rajukumar L, Baselga J, Terrones M, et al. Ultra-light carbon nanotube sponge as an efficient electromagnetic shielding material in the GHz range. physica status solidi (RRL) - Rapid Research Letters. 2014;8(8):698-704.

[10] Lu D, Mo Z, Liang B, Yang L, He Z, Zhu H, et al. Flexible, lightweight carbon nanotube sponges and composites for high-performance electromagnetic interference shielding. Carbon. 2018;133:457-63.

[11] Shen B, Li Y, Yi D, Zhai W, Wei X, Zheng W. Microcellular graphene foam for improved broadband electromagnetic interference shielding. Carbon. 2016;102:154-60.

[12] Micheli D, Pastore R, Vricella A, Morles RB, Marchetti M, Delfini A, et al. Electromagnetic characterization and shielding effectiveness of concrete composite reinforced with carbon nanotubes in the mobile phones frequency band. Materials Science and Engineering: B. 2014;188:119-29.

[13] Song Q, Ye F, Yin X, Li W, Li H, Liu Y, et al. Carbon Nanotube-Multilayered Graphene Edge Plane Core-Shell Hybrid Foams for Ultrahigh-Performance Electromagnetic-Interference Shielding. Advanced materials. 2017;29(31).

[14] Xi J, Li Y, Zhou E, Liu Y, Gao W, Guo Y, et al. Graphene aerogel films with expansion enhancement effect of high-performance electromagnetic interference shielding. Carbon. 2018;135:44-51.

[15] Zhang L, Alvarez NT, Zhang M, Haase M, Malik R, Mast D, et al. Preparation and characterization of graphene paper for electromagnetic interference shielding. Carbon. 2015;82:353-9.

[16] Shen B, Zhai W, Zheng W. Ultrathin Flexible Graphene Film: An Excellent Thermal Conducting Material with Efficient EMI Shielding. Advanced Functional Materials. 2014;24(28):4542-8.

[17] Paliotta L, De Bellis G, Tamburrano A, Marra F, Rinaldi A, Balijepalli SK, et al. Highly conductive multilayer-graphene paper as a flexible lightweight electromagnetic shield. Carbon. 2015;89:260-71.
[18] Liu Y, Zeng J, Han D, Wu K, Yu B, Chai S, et al. Graphene enhanced flexible expanded graphite film with high electric, thermal conductivities and EMI shielding at low content. Carbon. 2018;133:435-45.
[19] Zhou E, Xi J, Guo Y, Liu Y, Xu Z, Peng L, et al. Synergistic effect of graphene and carbon nanotube for high-performance electromagnetic interference shielding films. Carbon. 2018;133:316-22.
[20] Lu S, Shao J, Ma K, Chen D, Wang X, Zhang L, et al. Flexible, mechanically resilient carbon nanotube composite films for high-efficiency electromagnetic interference shielding. Carbon. 2018;136:387-94.
[21] Chaudhary A, Kumari S, Kumar R, Teotia S, Singh BP, Singh AP, et al. Lightweight and Easily Foldable MCMB-MWCNTs Composite Paper with Exceptional Electromagnetic Interference Shielding. ACS applied materials & interfaces. 2016;8(16):10600-8.
[22] Song W-L, Guan X-T, Fan L-Z, Cao W-Q, Wang C-Y, Zhao Q-L, et al. Magnetic and conductive graphene papers toward thin layers of effective electromagnetic shielding. Journal of Materials Chemistry A. 2015;3(5):2097-107.
[23] Ji K, Zhao H, Zhang J, Chen J, Dai Z. Fabrication and electromagnetic interference shielding performance of open-cell foam of a Cu–Ni alloy integrated with CNTs. Applied Surface Science. 2014;311:351-6.
[24] Ma J, Wang K, Zhan M. A comparative study of structure and electromagnetic interference shielding performance for silver nanostructure hybrid polyimide foams. RSC Advances. 2015;2015(5):65283-96.
[25] Jung J, Lee H, Ha I, Cho H, Kim KK, Kwon J, et al. Highly Stretchable and Transparent Electromagnetic Interference Shielding Film Based on Silver Nanowire Percolation Network for Wearable Electronics Applications. ACS applied materials & interfaces. 2017;9(51):44609-16.
[26] Choi HY, Lee T-W, Lee S-E, Lim J, Jeong YG. Silver nanowire/carbon nanotube/cellulose hybrid papers for electrically conductive and electromagnetic interference shielding elements. Composites Science and Technology. 2017;150:45-53.
[27] Shahzad F, Alhabeb M, Hatter CB, Babak Anasori, Hong SM, Koo CM, et al. Electromagnetic interference shielding with 2D transition metal carbides (MXenes). Science. 2016;353(6304):1137-40.
